# Antioxidant and Anti-Inflammatory Properties of Probiotic Candidate Strains Isolated during Fermentation of Agave (*Agave angustifolia* Haw)

**DOI:** 10.3390/microorganisms9051063

**Published:** 2021-05-14

**Authors:** Natalia C. Hernández-Delgado, Edgar Torres-Maravilla, Lino Mayorga-Reyes, Rebeca Martín, Philippe Langella, Ricardo Pérez-Pastén-Borja, María E. Sánchez-Pardo, Luis G. Bermúdez-Humarán

**Affiliations:** 1 Escuela Nacional de Ciencias Biológicas-Campus Zacatenco, Instituto Politécnico Nacional, Unidad Profesional Adolfo López Mateos, Zacatenco, Av. Wilfrido Massieu 399, Col. Nueva Industrial Vallejo, C.P. Alcaldía Gustavo A. Madero 07738, Mexico City, Mexico; nace_avril92@hotmail.com; 2 INRAE, AgroParisTech, Micalis Institute, Université Paris-Saclay, 78350 Jouy-en-Josas, France; edgar.torres-maravilla@inrae.fr (E.T.-M.); rebeca.martin-rosique@inrae.fr (R.M.); philippe.langella@inrae.fr (P.L.); 3 Departament of Biological Systems, Universidad Autónoma Metropolitana Xochimilco, Calzada del Hueso, 1100, Coyoacán 04960, Mexico City, Mexico; lmayorga@correo.xoc.uam.mx

**Keywords:** antioxidant, immunomodulation, *Lactobacillus*, probiotics, agave, IBD

## Abstract

Agave species are a source of diverse products for human use, such as food, fiber, and beverages, which include mezcal, a distilled beverage produced by spontaneous fermentation. Agave is an excellent source of high amounts of sugars, minerals, and phenolic compounds, which favor the growth of lactic acid bacteria (LAB) and yeast communities. In this work, 20 promising LAB strains with probiotic characteristics were isolated from the agave fermentation stage in mezcal production. The strains belonged to *Lactobacillus plantarum* (15), *Lactobacillus rhamnosus* (2), *Enterococcus faecium* (2), and *Lactococcus lactis* (1). These isolates were characterized for their resistance under gastrointestinal conditions, such as lysozyme, acid pH, and bile salts. In addition, the adherence of these LABs to human intestinal epithelial cells (Caco-2 and HT-29 cells) was tested in vitro and their antioxidant and immunomodulatory profile was determined using cellular models. *Lactobacillus rhamnosus* LM07 and *Lactobacillus plantarum* LM17 and LM19 strains were selected for their antioxidant properties, and their capacities in an oxidative stress model in intestinal epithelial cells IECs (Caco-2 and HT-29 cells) in the presence of hydrogen peroxide were evaluated. Interestingly, *Lactobacillus rhamnosus* LM07 and *Lactobacillus plantarum* LM17 and LM19 strains showed anti-inflammatory properties in TNF-α-stimulated HT-29 cells. Subsequently, bacterial strains exhibiting antioxidant and anti-inflammatory properties were tested in vivo in a mouse model with dinitrobenzene sulfonic acid (DNBS)-induced chronic colitis. Weight loss, intestinal permeability, and cytokine profiles were measured in mice as indicators of inflammation. One of the selected strains, *Lactobacillus plantarum* LM17, improved the health of the mice, as observed by reduced weight loss, and significantly decreased intestinal permeability. Altogether, our results demonstrate the potential of LAB (and lactobacilli in particular) isolated from the agave fermentation stage in mezcal production. *Lactobacillus rhamnosus* LM07 and *Lactobacillus plantarum* LM17 strains represent potential candidates for developing new probiotic supplements to treat inflammatory bowel disease (IBD).

## 1. Introduction

Probiotics are live microorganisms that confer a health benefit to the host when administered in adequate amounts [1]. For the identification and selection of potential candidate probiotic strains, important criteria are resistance to gastrointestinal passage (e.g., resistance to acid pH and bile salts), adhesion to the gut, and a beneficial effect on host health. Among the beneficial properties attributed to some probiotic strains, we can mention their anti-inflammatory and anti-proliferative effects against cancer cells, antagonism against pathogenic bacteria, cholesterol reduction, and antioxidant activity [2,3]. Reactive oxygen species (ROS) are associated with various gastrointestinal inflammatory and metabolic disorders, such as inflammatory bowel disease (IBD) and colorectal cancer (CRC) [4,5,6]. Interestingly, some probiotic strains, such *Enterococcus faecium*, *Oenococcus oeni, Lactobacillus* spp., and *Bifidobacterium* spp., have strong antioxidant capacity, as exhibited by their ability to reduce 2,2-diphenyl-1-picrylhydrazyl radical (DPPH), reduce antioxidant iron (FRAP), scavenge O_2_ radicals, and reduce iron ion (FE)-chelating activity. The beneficial antioxidant effects of LAB have been attributed to enzymes such as superoxide dismutase, catalase, glutathione peroxidase/GSH, and thioredoxin reductase/thioredoxin systems, which decrease the risk of ROS accumulation in the host during food intake and intestinal homeostasis. Nowadays, antioxidant activity is considered a functional property for the selection of candidate probiotic strains [7,8,9,10]. It is important to analyze the antioxidant effect using cellular, animal, and human trials to determine the efficiency and mechanism of antioxidative action.

Currently, there is great interest in the isolation of new probiotic LAB strains from unconventional sources, such as koumiss (a traditional fermented food produced from mare’s milk kefir in China) [11], kimchi (a traditional fermented and functional plant food from Korea) [12], and pulque (a traditional fermented beverage from *agave* spp. in Mexico) [13]. Agave plants are of great economic importance and represent numerous biotechnological advantages. Its high fructose, sugars, and fiber composition make agave useful for food and beverage production and even for biofuel production. Mezcal is a distilled alcoholic beverage fermented from agave, and its production has dramatically increased in the past years [14]. Although traditional fermentation occurs under stressful and uncontrolled environmental conditions [15], some microorganisms, such as certain LAB species, may adapt and survive. In the present work, new bacterial strains were isolated and characterized from agave must in mezcal production and their antioxidant (in vitro) and anti-inflammatory (*in vivo*) properties were determined to establish their probiotic potential.

## 2. Materials and Methods

### 2.1. Bacterial Sampling and Growth Conditions

Agave must samples were collected from mezcal production (during the month of August 2018) in the Santiago Matatlán, Tlacolula de Matamoros, and Macuilxóchilt de Ártigas Carranza localities (in the state of Oaxaca, southwest Mexico). The mezcal samples were mixed, diluted in phosphate buffer solution (PBS), and seeded on MRS agar (DIFCO, Mexico). The MRS plates were incubated at 37 °C for 48 h. Characteristic colonies were spread on the MRS plates, and successive passages were made to purify them. Fresh colonies were picked up to determine catalase production and perform Gram staining. Only catalase-negative and Gram-positive colonies were selected for further identification. Species confirmation was performed by 16S rRNA gene-targeted PCR using the following primers: fD1 (5′-ACGGCTACCTTGTTACGACTT-3′) and rP2 (5′-AGAGTTTGATCCTGGCTCAG-3′) [16].

Isolated candidate strains were cultured at 37 °C for 18 h in MRS broth (DIFCO, Mexico). The cells were then harvested by centrifugation at 4500× *g* for 10 min at 4 °C, rinsed twice with PBS (pH 7.4), and finally resuspended in PBS adjusted to an optical density of 1.0 at 600 nm [13].

The 20 isolates were compared to a LAB strain having well-documented *in vitro* and *in vivo* probiotic properties *Lactobacillus plantarum* Lp115 (ATCC SD5209) (Danisco, Brabrand, Denmark) [17,18].

### 2.2. Lysozyme, Low pH, and Bile Salt Resistance Tests

To determine the resistance of LAB isolated from agave to the gastrointestinal conditions they may encounter in vivo in the host, resistance to lysozyme (conditions simulating the human oral cavity), pH 2.5 (acidic condition of the human stomach), and 0.3% bile salts (simulation of small intestine environment) was evaluated in vitro models under conditions that mimic the digestive tract. Lysozyme and acid resistance were determined according to [19], and tolerance to bile salts was determined according to [20].

### 2.3. Adhesion and Antioxidant Assays

Cell surface hydrophobicity and adhesion to Caco-2 and HT-29 cell lines was determined according to the method previously described by Muñoz-Provencio et al. [21].

For antioxidant analyses, the scavenging activity of 2,2-diphenyl-1-picrylhydrazyl radical (DPPH) was determined according to Su et al. [22], and the scavenging measurement of hydroxyl radical (OH-) was determined according to the method proposed by Wang et al. [23].

### 2.4. Assays in TNF-α-Activated HT-29 Cells

The human colon carcinoma cell line HT-29 was seeded in 24-well culture plates in DMEM (Lonza, Switzerland) supplemented with 1% glutamine and 10% heat-inactivated FBS at 37 °C in a 10% CO_2_/air atmosphere. The culture medium was replaced daily. One day before bacterial co-culture (day 6), the culture medium was replaced with a medium containing 1% glutamine and 5% heat-inactivated FBS without antibiotics. The day of the co-culture, bacteria were added at a multiplicity of infection (MOI) of 1:40 in 50 µL of DMEM in a total volume of 500 µL. Cells were simultaneously activated with recombinant human TNF-α (5 ng/mL, Peprotech, New Jersey, NJ, USA) for 6 h at 37 °C in 10% CO_2_. After co-incubation, cell supernatants were stored at −80 °C until analysis of IL-8 content by ELISA (Biolegend, San Diego, CA, USA) [20].

### 2.5. Analysis of LAB Strains against Oxidative Stress in HT-29 Cells

Antioxidant indicators were measured according to Xing et al. [8] with some modifications. HT-29 cells were seeded in DMEM with antibiotics (penicillin–streptomycin, 50 units/mL) with 1% glutamine and 10% heat-inactivated fetal bovine serum (FBS; Gibco Invitrogen, Carlsbad, CA, USA) and incubated at 37 °C in 5% CO_2_. Cells were incubated in a flask for 5–8 days and then transferred (at 2 × 10^5^ cells/mL) to 24-well culture plates. Cells were cultured for 7 days until confluent monolayers were obtained. HT-29 cells were pretreated for 12 h with 64 mM butylated hydroxytoluene (BHT, Sigma-Aldrich, St. Louis, MO, USA), and subsequently, PBS or the different LAB strains were added and incubated in the presence of 2 mM H_2_O_2_ (Sigma-Aldrich, St. Louis, MO, USA) for an additional 6 h. After 18 h of incubation, the cells were rinsed with PBS and collected for measurement of antioxidant indicators. Total antioxidant status (TAS), superoxide dismutase (SOD), glutathione peroxidase (GPx), and catalase (CAT) were determined using the detection kit provided by RANDOX (County Antrim, UK). Lipid peroxidation products were measured by thiobarbituric-acid-reactive substances using the method described by Linden et al. [24].

Butylated hydroxytoluene (BHT) was used as a control antioxidant due to its ability to inhibit lipid peroxidation and decrease oxidative stress in many experimental models by restoring the status of cellular antioxidant enzymes [25].

### 2.6. Animal Experiments

Specific-pathogen-free male C57BL/6 mice (6–8 weeks) (Janvier, Le Genest Saint Isle, France) were maintained under normal breeding conditions at the animal care facilities of the Institut national de recherche pour l’agriculture, l’alimentation et l’environnement (IERP, INRAE, Jouy-en-Josas, France). All experiments were performed in accordance with European Community rules for animal care and were approved by the relevant local committee (Comethea; protocol number 16744-201807061805486 v2).

For colitis induction, a dinitrobenzene sulfonic acid (DNBS)-induced colitis protocol was performed according to Martin et al. with small modifications [26]. Briefly, mice were fully anesthetized by intraperitoneal (i.p.) injection of 150 μL of 0.1% ketamine (Imalgene 1000, Merial, France) and 0.06% xylazine (Rompun, Bayer HealthCare, Kansas, MO, USA), and a 3.5 catheter (French catheter, Solomon Scientific, Chicago, IL, USA) attached to a tuberculin syringe was inserted into the colon. Colitis was elicited by intrarectal injection through this tube of 200 mg kg^−1^ of DNBS solution (ICN Biomedical Inc., Santa Ana, CA, USA) dissolved in 30% ethanol (EtOH). Animals in the control group (no colitis) were given EtOH alone. The mice received 6% sucrose in drinking water for the initial 3 days after DNBS injection to prevent dehydration (DNBS period). Ten days after DNBS injection, 200 µL containing 1 × 10^9^ colony-forming units (CFU) of each bacterial strain or 200 µL of PBS were administered intragastrically every day for 10 days (gavage period). The study groups were as follows: control group without colitis (EtOH-PBS), control group with colitis (DNBS-PBS), *Lactobacillus rhamnosus* LM07 (DNBS-LM07), *Lactobacillus plantarum* LM17 (DNBS-LM17), and LM19 (DNBS-LM19). Colitis was reactivated 21 days after the first DNBS injection (recovery period) with a second injection of 100 mg/kg of DNBS solution [26].

### 2.7. Macroscopic Scores

The mice were sacrificed by cervical dislocation, and the abdominal cavity was opened. The colon and small intestine were removed and opened longitudinally, and visible damage was immediately assessed. Macroscopic scores were documented using a system previously described for DNBS colitis [26]. Briefly, macroscopic criteria (assessed on a scale of 0–9) included macroscopic mucosal damage (such as ulcers, thickening of the colon wall, presence of adhesions between the colon and other intra-abdominal organs), stool viscosity (as an indicator of diarrhea), and the presence of hyperemia.

### 2.8. In Vivo Permeability Assay (FITC) and Myeloperoxidase Activity

Intestinal barrier function was assessed using fluorescein isothiocyanate–dextran (FITC-dextran). The mice were administered FITC-dextran (0.6 mg/g body weight, molecular weight 3000–5000 Da; Sigma-Aldrich, St. Louis, MO, USA) intragastrically 3.5 h before sacrifice. Blood samples were obtained from the retro-orbital venous plexus, and the intensity of fluorescence in serum was calculated using a microplate reader (Tecan, Lyon, France). The FITC-dextran concentration was calculated from a standard curve obtained from a serial dilution of FITC-dextran [27].

Myeloperoxidase (MPO) activity, used as a marker of neutrophil infiltration, was measured by a modified version of the method described by Bradley et al. [28]. A centimeter-long fragment was obtained from the distal colon and homogenized (50 mg/mL) in cold 50 mM potassium phosphate buffer (pH 6) containing 5% hexadecyltrimethylammonium bromide (Sigma-Aldrich, Lyon, France) and hydrogen peroxide (H_2_O_2_, Sigma-Aldrich, Lyon, France). The colorimetric reaction was followed by measuring absorbance with a plate reader (Tecan, Lyon, France). MPO activity is represented in units per milligram of wet tissue, with 1 unit being the activity required to transform 1 mM H_2_O_2_ into water in 1 min at room temperature.

### 2.9. Statistics

Statistical analyses were performed using GraphPad (GraphPad Software, San Diego, CA, USA). Analysis of normality and variance was performed using the Shapiro–Wilk normality test. For normal samples with equal variances, two-way ANOVA was performed. Multiple comparisons were performed using Tukey’s test. For non-normal samples and unequal variances, within-group nonparametric tests were performed (Kruskal–Wallis test), multiple comparisons were performed using Dunn’s test, and *p <* 0.05 was considered to be a statistically significant level.

## 3. Results

### 3.1. Strain Identification

Twenty Gram-positive, catalase-negative bacterial strains were isolated from the mezcal batch samples according to 16S gene amplification (Table 1). *Lactobacillus plantarum* (15 strains) was the predominant isolated species (from the three different regions in the samples), followed by *Lactobacillus rhamnosus* (2 strains), *Enterococcus faecium* (2 strains), and *Lactococcus lactis* (1 strain). Of note, samples from the region of Tlacolula de Matamoros showed the greatest variety of isolated strains.

### 3.2. Lysozyme, pH, and Bile Salt Tolerance of the Isolated Bacteria

In this study, the resistance of all isolated strains to the conditions they may encounter in the digestive tract (such as lysozyme, low pH, and bile tolerance) was tested in vitro using *L. plantarum* Lp115 as the reference probiotic strain (Table 2). In the first assay (in the presence of lysozyme), all strains showed a survival rate higher than 30% after 3 h of incubation. In all cases, there were significant differences with the reference strain Lp115. *Enterococcus faecium* LM15 showed the highest survival rates (77.06%) to lysozyme, followed by *L. plantarum* LM02 and *L. rhamnosus* LM07 (66.22%). The second challenge was exposure to low pH to mimic stomach conditions; only eight of the isolated strains showed better survival rates than *L. plantarum* Lp115 (38.30% survival). *L. plantarum* LM20 showed the highest survival rates (75.86%), followed by *L. plantarum* LM19 (68.84%), LM14 (61.73%), and LM17 (53.93%). Finally, the bacteria were subjected to bile salts. Half of the strains (10) showed superior survival rates (≥50%) to bile salts, with *L. plantarum* LM19 and *E. faecium* LM15 standing out with ~80% survival after exposure to bile salts.

### 3.3. Cell Culture Methods and Biochemical Characterization of the Isolated Strains

**Figure 1:**
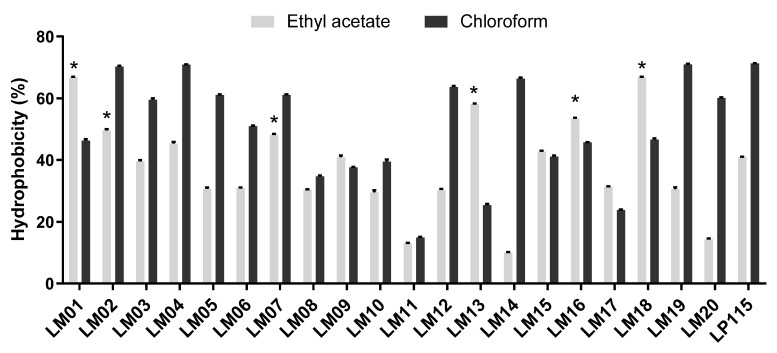
Cell surface properties of lactic acid bacteria (LAB) isolated during fermentation of agave by affinity to solvents. Results are presented by the mean ± SEM; * indicates significant (*p* < 0.05) superiority to *L. plantarum* Lp115 values.

The hydrophobic properties of all strains to ethyl acetate and chloroform are shown in Figure 1. All strains tested showed an affinity to chloroform (non-polar solvent) rather than to ethyl acetate (electron acceptor, higher polarity). Remarkably, *L. plantarum* LM04 was the strain showing the highest affinity rates of 70% and 45% to ethyl acetate and chloroform, respectively.

**Figure 2:**
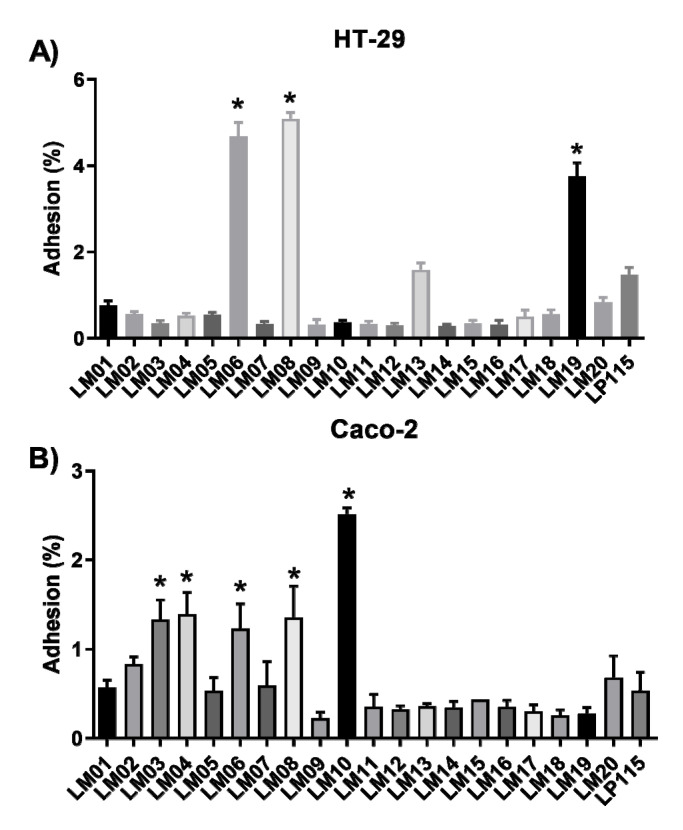
In vitro adhesion of lactic acid bacteria (LAB) isolated from agave to HT-29 (**A**) and Caco-2 cells (**B**). Results are the mean ± SEM from three independent experiments. * Indicates a significant difference as compared to *L. plantarum* Lp115 (*p <* 0.05).

Next, we tested the adhesion of the bacterial strains to intestinal epithelial cells: Caco-2 (ATCC^®^ HTB-37™) and HT-29 (ATCC^®^ HTB38™). In general, the binding values to Caco-2 and HT-29 cells were lower: ~0.2% and ~0.4%, respectively (Figure 2). However, the values for LM06, LM08, and LM19 strains were significantly superior (≥4% adhesion) to the values obtained for *L. plantarum* Lp115 (Figure 2A). Although lower adhesion values were observed in Caco-2 cells, five of the strains (LM03, LM04, LM06, LM08, LM10) obtained percentage values of adhesion to the reference strain (Figure 2B).

**Figure 3:**
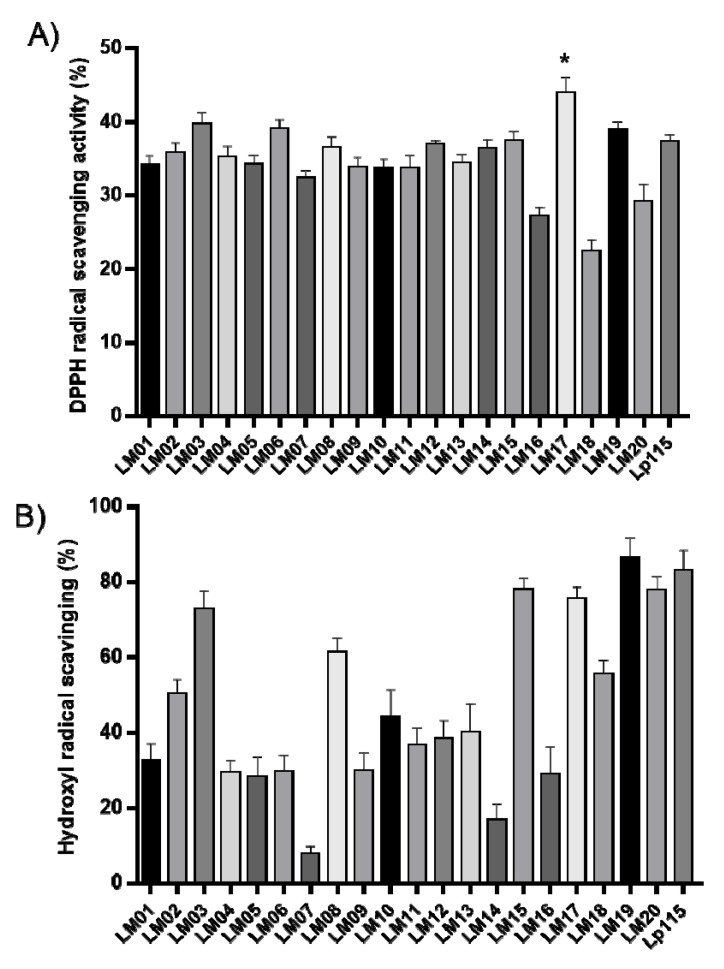
DPPH radical-scavenging (**A**) and hydroxyl radical-scavenging (**B**) activities of lactic acid bacteria (LAB)**.** Results are the mean from three independent experiments; error bars indicate the mean ± SEM. * indicates a significant difference as compared to *L. plantarum* Lp115 (*p* < 0.05).

Then, we performed antioxidant assays on the strains. For this, the hydroxyl radical and DPPH radical-scavenging capacity of the *Lactobacillus* strains are shown in Figure 3. The percentage inhibition values of the DPPH radical ranged from 27.12% (LM16) to 43.99% (LM17); moreover, the *L. plantarum* strain LM17 significantly (*p <* 0.05) exceeded the activity of the commercial strain *L. plantarum* Lp115 (37.39%) (Figure 3A). In contrast, in the hydroxyl radical-quenching assay, the range was 7.83% (LM07) to 86.64% (LM19) (Figure 3B). Although none of the strains outperformed the control Lp115, strains LM03, LM15, LM17, LM19, and LM20 showed levels of sequestration activity similar to that of Lp115 (Figure 3B).

### 3.4. Screening of Bacterial Strains in HT-29 Cells Stimulated with TNF-α

**Figure 4:**
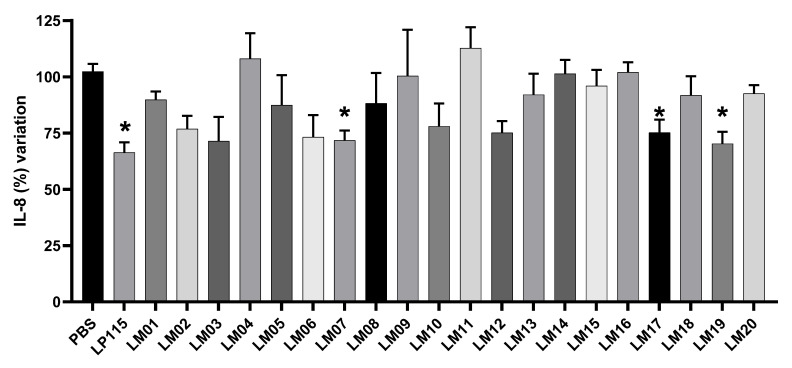
Effect of LAB isolated from agave samples on IL-8 production on HT-29 cells. Results are presented as means ± SEM. * indicates a significant difference as compared to the PBS group (*p <* 0.05).

We also determined the ability of our LAB strains to block IL-8 secretion by TNF-α-stimulated HT-29 cells. Since IL-8 is considered an important inflammatory mediator, candidate bacteria that increase its secretion will be considered to have proinflammatory properties, whereas those that inhibit its secretion will be considered to have anti-inflammatory properties. Our data show that *L. plantarum* strains LM17 and LM19 and *L. rhamnosus* LM07 significantly (*p* < 0.05) blocked the secretion of IL-8 production by 33–34% (Figure 4).

### 3.5. Role of LAB against Oxidative Stress

The potential protective effect of LAB isolated from *Agave* against oxidative stress induced in HT-29 cells is shown in Table 3. Compared to cells treated with H_2_O_2_ alone, the antioxidant BHT reduced the MDA concentration 5-fold in contrast to the increase in the total antioxidant status (TAS). In addition, the activity of SOD, GPx, and CAT enzymes was significantly increased by BHT. Similarly, co-culture of strains LM07, LM17, and LM19 with HT-29 cells recovered enzyme activity, although to a lesser extent than the reference antioxidant.

### 3.6. Anti-Inflammatory Effects of LAB Strains Isolated from Agave

**Figure 5:**
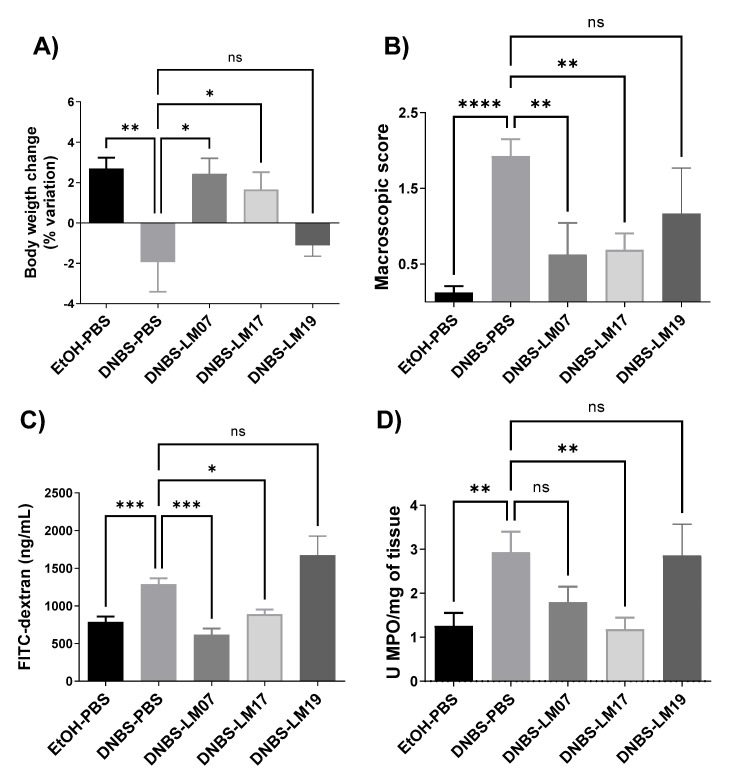
Effect of *Lactobacillus* strains isolated from agave samples in a DNBS chronic colitis model. (**A**) Body weight variation of mice, (**B**) macroscopic score, (**C**) gut permeability, and (**D**) MPO/mg of tissue. Results are presented as the means ± SEM. ns: not significant, * Indicates a significant difference as compared to the DNBS group (*p <* 0.05, ** *p <* 0.01, *** *p <* 0.001, **** *p* < 0.0001).

Finally, we selected the two most promising anti-inflammatory strains (based on the results described above), *L. rhamnosus* LM07 and *L. plantarum* LM17 and LM19, to evaluate their protective effects in a DNBS-induced murine colitis model. As shown in Figure 5, the weight loss of the mice was affected by DNBS, resulting in a 5% weight loss at the endpoint. In contrast, the EtOH-PBS group gained 5% (Figure 5A). Interestingly, administration of *L. rhamnosus* LM07 and *L. plantarum* LM17 protected against weight loss, normalizing body weight to the level of that of the control group. Furthermore, both strains significantly (*p <* 0.05) reduced the total macroscopic score of colon visual damage (Figure 5B) and, at the same time, the intestinal hyperpermeability of the mice (Figure 5C). However, only *L. plantarum* LM17 reduced myeloperoxidase (MPO) levels to that of healthy animals (Figure 5D).

## 4. Discussion

The agave fermentation process involves a predominant community of lactobacilli (48.9%), in addition to *Pediococcus, Weissella,* and *Bacillus*, varying from one distillery to another and by the season of the year in which it was sampled [15,29]. Interestingly, *L. rhamnosus*, a facultative heterofermentative bacterium, has been isolated from different mucous membranes of the human body and several artisanal and processed dairy products, such as cheeses [30]. This reflects that traditional fermentation, which occurs in open air, allows progressive modification of the microbial community. A total of 20 catalase-negative strains belonging mainly to the genus and species *L. plantarum* and *L. rhamnosus* were isolated in this work.

The profile of probiotic candidates that viably transit through the small and large intestines and, in some specific cases, colonize the host was researched during this study. Gram-positive lactobacilli can resist the action of the enzyme lysozyme (a nonspecific defense enzyme secreted in the mouth) due to the presence of peptidoglycan [31]. Although the bacteria evaluated here present peptidoglycan, only strains LM19 and LM20 showed high tolerance to lysozyme. However, the rest of the strains tested showed survival rates similar to those of the reference strain Lp115, suggesting the presence of additional mechanisms. Biofilm production confers adhesion properties and resistance against gastric acidity to the lactobacilli that produce them [32]. Furthermore, the membrane proton pump F0F1-ATPase keeps protons below the intracellular threshold, preventing lethal cell damage [33]. Thus, it is possible that strains LM17, LM19, and LM20, having a survival rate of 59% to an acidic medium, compared to the reference strain *L. plantarum* Lp115, may stand out through a combination of these mechanisms. In the small and large intestines, a shift in gastric pH occurs and bile salts’ detergent action limits bacterial viability. Lactobacilli and bifidobacteria recovered from intestinal contents present the enzyme bile salt hydrolase, which converts primary bile acids into secondary (less active) bile salts [30]. Therefore, of all strains evaluated, the strain LM19 survived by 50% in the bile salt resistance test. Altogether, strains LM17, LM19, and LM20 showed the highest survival potential in the GIT.

Adhesion to the intestinal mucosal surface is essential for the colonization of LAB in the GIT, resulting in antagonistic activity against pathogens, regulation of the immune system, and an increase in the primary host’s defenses, among many other functions. The microbial adhesion test to solvents reveals the chemical nature of the microbial surface. Our results revealed that only strains LM04 and LM19 showed a higher affinity to acidic solvents (chloroform) than to basic solvents (ethyl acetate). These results are similar to those reported by Dlamini, evaluating strains of *Lactobacillus* spp. and *Streptococcus* spp. [34]. The abundant functional groups on membrane surface components, such as COO- and -HSO_3_, act as a Lewis acid, a strong electron donor [35]. In contrast, LM06, LM08, and LM19 were significantly more adherent; however, the strains exhibited low adhesion, and these results agree with those reported by Yang et al. and Torres-Maravilla et al. [13,36]. The adhesion capacity of these strains could explain the specific binding capacity of probiotic strains, which involves surface proteins such as fibronectin and sortases [37].

IECs represent the first point of contact of the host with bacteria, resulting in a stimulation of the immune system and an induction of cytokine production. The ability of a probiotic candidate bacterium to modulate the immune response is an important criterion. Here, we tested the ability of strains isolated from agave to regulate IL-8 in the TNF-α-induced HT29 cell model. We found that *L. rhamnosus* LM07 and *L. plantarum* strains LM17 and LM19 inhibited IL-8 secretion. Our results are consistent with similarly studies performed with plant-derived *Lactobacillus* strains [13,38]. HT-29 cells with adherent bacteria remain hyporeactive to TNF-α and secrete less IL-8. A cell wall compound lipoteichoic acid (LTA) has been described in *Lactobacillus* species, which induces the suppression of IL-8 secretion and the p38, NF-κB, and ERK signaling pathways [39]. Soluble bioactive molecules have also been shown to be secreted by *Lactobacillus rhamnosus* R0011, down-regulating proinflammatory chemokine production of human HT-29 cells simulated with proinflammatory signaling. Furthermore, LAB–host communication has been shown to mediate the regulation of the expression of crucial immune response signaling genes, such as NF-κB and MAPK, among others [40,41].

According to our results obtained *in vitro* assays, all the LAB studied in this work display an antioxidant capacity comparable to that of BHT, since they decrease the levels of DPPH and hydroxyl radicals. *Lactobacillus* species produce non-enzymatic antioxidant molecules such as proteins, polysaccharides, and peptides. In addition, their metabolism features antioxidant systems such as thioredoxin/thioredoxin reductase and glutathione/glutathione reductase, which efficiently neutralize ROS [42,43,44].

It was proved that agave isolates LM07, LM17, and LM19 protect HT-29 cells against hydrogen-peroxide-induced damage, since they increased the enzymatic activities of superoxide dismutase, glutathione peroxidase, and catalase. Aerobic and microaerophilic lactobacilli such as those isolated here possess enzyme systems such as NADH oxidase [45], pyruvate oxidase [46], and lactate oxidase [9], which consume oxygen, reducing its availability and limiting the production of superoxide anion and hydrogen peroxide. However, Mn^2+^-dependent superoxide dismutase, the most crucial antioxidant enzyme in lactobacilli, consumes the superoxide anion and produces hydrogen peroxide [36,47]. Kullisaar et al. [48] reported that *L. fermentum* E-3 and E-18 expressed Mn-SOD, capable of resisting oxidative stress. Finally, hydrogen peroxide, which is regularly degraded by the action of catalase in most organisms, can be used as a source of hydrogen peroxide [48]. Considering that strains evaluated here were catalase negative, the recovery of CAT activity in the present work is worth noting. The efficacy of different probiotic strains in various mouse models of DNBS-induced colitis has been documented to study the pathogenesis of IBD. IBD produced by increased reactive oxygen species (ROS) and inflammation has been shown to play a role in the disruption of the intestinal epithelium [49].

We found that *L. rhamnosus* LM07 and *L. plantarum* LM17 improved some colitis symptoms in the DNBS-induced colitis murine model. Strains LM07 and LM17 decreased intestinal permeability, as observed by reduced FITC-dextran levels in serum samples, prevention of weight loss, and a decrease in MPO levels in colonic tissue samples. According to Yoda et al., probiotics prove to be an effective barrier against diseases caused by pathogens and chemical agents [50]. For example, *L. rhamnosus* LGG and its proteins p40 and p75 reversed the injury caused by dextran sodium sulfate (DSS) in a mouse model of colitis, or more recently, a novel secreted protein HM0539 from LGG exhibited a potent protective effect on the intestinal barrier [51]. Comparable findings reversing the permeability characteristic of inflammation occurred with *L. plantarum* strains DSM 9843(2099v) and *L. reuteri* R2LC in a methotrexate-induced mouse model of colitis [52]. The strains studied here, LM07 and LM17, reduced neutrophilic thrombocyte infiltration, as observed by decreased MPO activity. A characteristic process of inflammation is the degranulation of neutrophils, which release MPO into the phagolysosomes. Therefore, the activity of this enzyme has been used as a biomarker of early inflammation [53]. Notably, administration of strain LM19 did not reduce inflammatory markers in the mouse model, whereas strain LM17 effectively attenuated some symptoms in the DNBS-induced colitis model. Since the biological effect of each isolate (including those of the same genus and species) is strain dependent, careless generalizations should not be made [54]. Therefore, the assessment of whether a strain is probiotic should be made on an individual basis.

In conclusion, our results indicate that strains *L. rhamnosus* LM07 and *L. plantarum* LM17 have probiotic potential for use in the context of IBD and the importance of *Agave* spp. as a source of microorganisms with beneficial effects on intestinal health. However, the mechanism underlying their probiotic effect still needs to be elucidated, and further studies are necessary to validate these properties in other in vivo models.

## Figures and Tables

**Table 1:** List of lactic acid bacteria (LAB) isolated from agave-fermented samples from Macuilxóchilt de Ártigas, Tlacolula de Matamoros, and Santiago Matatlán in Oaxaca, México.

Code	Microorganism	Region	16S rRNA (%)
LM01	*Lactobacillus plantarum*	Macuilxóchilt de Ártigas	98.8
LM02	*Lactobacillus plantarum*	Macuilxóchilt de Ártigas	99
LM03	*Lactobacillus plantarum*	Macuilxóchilt de Ártigas	95.7
LM04	*Lactococcus lactis ssp. lactis*	Tlacolula de Matamoros	99
LM05	*Lactobacillus plantarum*	Tlacolula de Matamoros	97.4
LM06	*Enterococcus faecium*	Santiago Matatlán	99
LM07	*Lactobacillus rhamnosus*	Tlacolula de Matamoros	100
LM08	*Lactobacillus plantarum*	Tlacolula de Matamoros	98.1
LM09	*Lactobacillus plantarum*	Macuilxóchilt de Ártigas	97.5
LM10	*Lactobacillus plantarum*	Macuilxóchilt de Ártigas	97.4
LM11	*Lactobacillus plantarum*	Tlacolula de Matamoros	96.7
LM12	*Lactobacillus plantarum*	Santiago Matatlán	96
LM13	*Lactobacillus plantarum*	Tlacolula de Matamoros	97
LM14	*Lactobacillus plantarum*	Tlacolula de Matamoros	97.6
LM15	*Enterococcus faecium*	Macuilxóchilt de Ártigas	99
LM16	*Lactobacillus rhamnosus*	Tlacolula de Matamoros	99
LM17	*Lactobacillus plantarum*	Tlacolula de Matamoros	99.9
LM18	*Lactobacillus plantarum*	Tlacolula de Matamoros	97
LM19	*Lactobacillus plantarum*	Santiago Matatlán	99
LM20	*Lactobacillus plantarum*	Macuilxóchilt de Ártigas	99

**Table 2:** Tolerance to gastrointestinal tract conditions of lactic acid bacteria (LAB) isolated from agave fermentation: 0, 90, and 180 min of incubation.

Isolate	Lysozyme			pH 2.5				0.3% Bile Salt			
	0	180	Survival (%)	0	90	180	Survival (%)	0	90	180	Survival (%)
	Log CFU/mL			Log CFU/mL				Log CFU/mL			
LM01	9.19	8.93	54.70 *	9.19	9.09	8.77	38.10	9.19	9.10	8.92	53.68
LM02	9.14	8.97	67.15 *	9.31	8.56	8.33	10.46	9.31	9.23	8.70	24.84
LM03	9.35	8.97	41.59 *	9.35	9.04	8.74	24.70	9.35	9.11	8.92	37.50
LM04	9.32	9.13	64.76 *	9.33	9.02	8.92	38.58	9.33	9.23	9.00	45.99
LM05	9.39	8.97	38.25 *	9.35	9.03	8.82	28.91	9.35	9.21	9.02	46.31
LM06	9.25	8.80	35.58 *	9.39	8.89	8.56	14.52	9.39	9.28	8.72	21.24
LM07	9.18	9.00	66.22 *	9.29	8.71	8.44	13.95	9.29	9.21	8.75	28.57
LM08	9.31	8.99	47.57 *	9.45	9.18	8.97	33.10	9.45	9.28	8.94	31.19
LM09	9.38	9.00	41.05 *	9.36	9.08	8.83	29.82	9.36	9.24	8.88	33.04
LM10	9.42	8.90	29.80 *	9.35	9.13	9.00	44.35	9.35	9.27	8.95	39.58
LM11	9.43	9.15	52.24 *	9.21	8.76	8.66	27.64	9.21	9.15	8.97	57.32 *
LM12	9.25	8.95	50.19 *	9.25	8.94	8.73	30.68	9.25	9.13	8.85	40.53
LM13	9.15	8.85	49.77 *	9.31	9.09	9.05	55.56 *	9.31	9.14	9.06	55.88 *
LM14	9.19	8.82	42.31 *	8.73	8.64	8.52	61.73 *	8.73	8.62	8.52	61.73 *
LM15	9.27	9.16	77.06 *	8.94	8.73	8.51	36.36	8.94	8.94	8.85	81.06 *
LM16	9.33	9.14	64.78 *	8.86	8.60	8.50	43.52	8.86	8.64	8.51	44.44
LM17	9.04	8.45	25.45	9.30	9.12	9.07	59.93 *	9.3	9.22	8.93	43.43
LM18	9.36	8.90	34.20 *	9.26	9.12	8.84	38.15	9.26	8.99	8.85	39.63
LM19	9.32	9.06	55.45 *	8.96	8.93	8.80	68.84 *	8.96	8.93	8.90	86.23 *
LM20	9.35	9.06	51.79 *	9.06	9.00	8.94	75.86 *	9.06	8.94	8.88	64.94 *
Lp115	9.44	8.88	27.74	8.97	8.92	8.56	38.30	8.97	8.78	8.67	49.65

* Indicates a significant difference as compared to *L. plantarum* Lp115 (*p <* 0.05). LAB: lactic acid bacteria. CFU/mL: colony-forming-unit per milliliter.

**Table 3:** Effects of *Lactobacillus* co-cultured with colonic cells HT-29 under oxidative stress with hydrogen peroxide.

	2 mM Hydrogen Peroxide				
Group	MDA nmol/mg Protein	TAS mmol/mg Protein	SOD U/mg Protein	GPx U/mg Protein	CAT U/mg Protein
**PBS**	5.20 ± 0.03 ^b,c^	0.16 ± 0.001 ^b,c^	18.45 ± 0.0003 ^b,c^	1.15 ± 0.08 ^b,c^	0.87 ± 0.11 ^b,c^
**BHT**	1.10 ± 0.02 ^a,c^	2.24 ± 0.03 ^a^	28.39 ± 0.001 ^a,c^	5.59 ± 0.05 ^a^	4.57 ± 0.06 ^a,c^
**Lp115**	1.28 ± 0.07 ^a,b^	1.92 ± 0.65 ^a^	26.64 ± 0.49 ^a,b^	4.99 ± 0.06 ^a^	3.67 ± 0.12 ^a,b^
**LM07**	2.11 ± 0.06 ^a,b,c^	1.40 ± 0.12 ^a,b,c^	22.36 ± 0.005 ^a,b,c^	4.38 ± 0.10 ^a,b,c^	3.36 ± 0.37 ^a,b^
**LM17**	2.49 ± 0.11 ^a,b,c^	2.10 ± 0.08 ^a^	23.15 ± 0.003 ^a,b,c^	4.11 ± 0.11 ^a,b,c^	3.95 ± 0.21 ^a^
**LM19**	2.73 ± 0.02 ^a,b,c^	1.73 ± 0.14 ^a,b^	23.42 ± 0.001 ^a,b,c^	4.39 ± 0.21 ^a,b,c^	3.25 ± 0.12 ^a,b^

Results are the mean ± SEM from three independent experiments. *n =* 3. MDA: malondialdehyde; TAS: total antioxidant status; SOD: superoxide dismutase; GPx: glutathione peroxidase; CAT: catalase; PBS: phosphate buffer saline; BHT: butylated hydroxytoluene. Results with common superscript letters do not differ significantly (*p* < 0.05): *a* vs. PBS; *b* vs. BHT, and *c* vs. Lp115.
